# The up‐regulated hsa‐circRNA9102‐5 may be a risk factor for essential hypertension

**DOI:** 10.1002/jcla.23339

**Published:** 2020-05-23

**Authors:** Shuying Zheng, Xin He, Jihan Sun, Qiang Li, Tao Zhang, Lina Zhang

**Affiliations:** ^1^ Department of Preventative Medicine Zhejiang Provincial Key Laboratory of Pathological and Physiological Technology Medicine School of Ningbo University Ningbo China; ^2^ Department of Clinical Medicine Medicine School of Ningbo University Ningbo China; ^3^ Department of Radiology Yinzhou People's Hospital Ningbo China; ^4^ Department of Chronic Disease Control and Prevention Ningbo Municipal Center for Disease Control and Prevention Ningbo China

**Keywords:** biological function, biomarker, circular RNA, essential hypertension, gene expression

## Abstract

**Background:**

The present study was aimed to investigate the expression levels of circular RNAs (circRNAs) in the peripheral blood of essential hypertension (EH) patients and healthy controls (HC). On this basis, we tried to explain the possible role of circRNAs in the progression of EH and their potential as diagnostic biomarkers of EH.

**Methods:**

First, we analyzed the differentially expressed circRNAs in peripheral blood obtained from the finished microarray analysis and selected four circRNAs under strict standards. Then, quantitative real‐time polymerase chain reaction (qRT‐PCR) was performed to measure the expression levels of the selected circRNAs in a total of 192 blood samples, consisting of 96 HC and 96 diagnosed EH patients. Bioinformatics prediction of the target microRNAs (miRNAs) was performed for differentially expressed circRNAs, and the circulating vascular‐related miRNAs were selected for qRT‐PCR analysis to determine their expression levels.

**Results:**

Hsa‐circRNA9102‐5 (11.7 ± 1.06 vs 12.13 ± 1.11, *P* = .007) was up‐regulated in the patients group which was diagnosed with EH, as compared to the HC group, and was involved in the regulation of EH by sponging hsa‐miR‐150‐5p. The area under the ROC curve (AUC) of the model was 0.620, using hsa‐circRNA9102‐5 as an independent predictor. Furthermore, the AUC was increased to 0.728 when hsa‐circRNA9102‐5 was combined with hsa‐miR‐150‐5p and multiple other factors, as a combined predictor.

**Conclusions:**

The present results suggested that hsa‐circRNA9102‐5 may have played a crucial role in the development of EH by sponging hsa‐miR‐150‐5p, which showed great potential as a novel target.

## 1 INTRODUCTION

Essential hypertension (EH) is not only a chronic multi‐factorial disease, but also a major public health problem worldwide. EH, a dominating risk factor for an early death or a disability, affects the urban and rural regions around the world. According to the World Health Organization (WHO), the worldwide prevalence of hypertension will be 29.2% by 2025, and the majority will be governed by EH.^1^ The initial symptoms of EH are insidious, and the patients often suffer from various serious complications as the disease progresses to advanced stages. Hence, there is a pressing need for early diagnosis and intervention. Although some environmental risk factors have been reported successfully, the genetic pathogenesis remains unclear. Some researchers have investigated the epigenetic mechanism of DNA methylation in the development of EH,^2^, ^3^ and others have explored the association between non‐coding RNAs and EH.^4^ Researchers have gradually found that a number of microRNAs (miRNAs) have been closely related to EH because they are easy to obtain for research.

Unlike linear RNAs, circRNAs are considered as a special type of closed circular non‐coding RNA without a 5′ cap or a 3′ tail.^5^ The major structural features of the circRNAs have been reported to be conserved across species, stable, and free of RNase R‐mediated degradation.^6^ Jeck et al^7^ indicated that the abundance of circRNAs was more than 10 times of the associated linear mRNA. An important biological function of circRNAs is that they can be used as a miRNA sponge to suppress the ability of the miRNA to bind to its downstream mRNA target.^8^, ^9^

Researchers have found that circRNAs play important roles in many cardiovascular diseases (CVD) including myocardial infarction, heart failure, atherosclerosis, and myocardial fibrosis via sponging miRNA.^10^ Besides regulating gene expressions through miRNAs, researchers have begun to consider other functional pathways of the circRNAs. For example, the CircFoxo3 directly suppresses the function of the target proteins to promote a myocardial cell senescence.^11^ However, few reports have been published on the EH‐related circRNAs, either in terms of their mechanism or their roles as diagnostic biomarkers.

In this study, we analyzed differentially expressed circRNAs in the peripheral blood obtained from a finished microarray analysis and confirmed the selected circRNAs in larger independent cohorts. We combined miRNAs to build a joint network to explore the possible role of circRNAs in EH. For future research, circRNAs may not only be regarded as a biomarker of cardiovascular risk, but also as a therapeutic target for improving the cardiovascular status in adults suffering from high blood pressure.

## 2 MATERIALS AND METHODS

### 2.1 Collection of samples

A total of 192 participants in this study, consisting of 96 EH patients and 96 sex‐matched and age(±3 years)‐matched HC, were collected from the Yinzhou People's Hospital (Ningbo, China) from October 2018 to July 2019. All the participants in this study were determined according to the unified inclusion and exclusion criteria, and a written informed consent was signed by each participant before participation. In this study, the EH patients were defined as the individuals whose systolic blood pressure (SBP) was ≥140 mm Hg and/or diastolic blood pressure (DBP) was ≥90 mm Hg, in at least three consecutive measurements.^12^ The HC group had SBP < 130 mm Hg and DBP < 85 mm Hg, and had never been diagnosed with high blood pressure by any reliable health agency. All the controls and EH patients had a similar occupation of an office clerk and their ages ranged from 35 years to 75 years. None of the subjects had any history of genetic hypertension and related drug treatments, and were also free of overt cardiovascular diseases, kidney diseases, drug abuse, diabetes mellitus, or other serious diseases, as evaluated by their medical history and fasting blood biochemistry results. The fasting blood samples of the participants were collected from the anterior cubital vein into ethylenediaminetetraacetic acid (EDTA) anticoagulant Vacutainers and were immediately stored at −80°C. The total RNA was extracted within a week of storage.

The present study, including the recruitment of participants from the affiliated hospital, was approved by the Human Research Ethics Committee of the Ningbo University (Ningbo, China).

### 2.2 Detection of relevant indices and extraction of total RNA

The blood biochemical index data was obtained by using an AU2700 High‐Volume Chemistry‐Immuno Analyzer (Olympus). Physical examination, including height, weight, blood pressure, and behavioral lifestyle including smoking and drinking, were conducted by an experienced medical staff. Body mass index (BMI) was measured as weight (kg) divided by the height squared (m^2^).

A total RNA extraction kit (BioTeke) was used to extract total RNA from the peripheral blood samples in accordance with the manufacturer's instructions. Agarose gel electrophoresis was performed to detect the RNA integrity. The total RNA purity and quantity was measured using the Multiskan™ GO Microplate Spectrophotometer (Thermo Scientific).

### 2.3 Candidate circRNAs and their target miRNAs prediction

We previously analyzed peripheral blood circRNAs between 5 diagnosed EH patients and 5 healthy controls using a microarray analysis and we found a total of 287 circRNAs were differentially expressed between the two groups including 209 up‐regulated circRNAs and 78 down‐regulated circRNAs.^13^ For subsequent functional analysis to obtain the potential biomarkers for clinical application, the circRNAs were selected from the up‐regulated exonic circRNAs (ecircRNAs) using the following filtering criteria: fold change >2.0 and *P* < .01. Then, hsa_circ_0093587 and hsa‐circRNA9102‐5 were included in this study. To explore the potential function of circRNAs in the pathogenesis of EH, bioinformatics prediction of the target miRNAs was performed for the circRNAs which were identified as differentially expressed, using miRanda and RNA22 software. Only when a circRNA‐miRNA sequence matching was predicted by both software, to have at least one match, it was considered as a candidate miRNA. Circulating vascular‐related miRNAs were selected from the candidate miRNAs for subsequent validation. Finally, using the predictive software we found that the vascular‐related miRNA hsa‐miR‐150‐5p had more than one binding site with hsa‐circRNA9102‐5.

### 2.4 Functional enrichment analysis of target miRNAs for differentially expressed circRNAs

In this study, the target genes of miRNA were predicted by using three bioinformatics databases, including the TargetScan, the DIANA‐microT, and miRanda. According to the different algorithms of each database, the predicted target genes were also different and the number of predicted target genes reached thousands in their count. We took their intersection to reduce the false‐positive rate. After that, the Gene Ontology (GO) and the Kyoto Encyclopedia of Genes and Genomes (KEGG) (http://kobas.cbi.pku.edu.cn/index.php) were used to annotate and enrich the functions of the genes in the pathways.

### 2.5 Quantitative real‐time polymerase chain reaction (qRT‐PCR) analysis

The total RNA extracted from the peripheral blood samples was reverse transcribed when the A260/A280 value was within the range of 1.8‐2.1, as measured by a NanoDrop 2000 instrument (Thermo Scientific), and the integrity of RNA met the requirements. CircRNA, in the total RNA sample, was reverse transcribed to a complementary DNA (cDNA) using the GoScript RT System (Promega). MiRNA, in the total RNA sample, was reverse transcribed to cDNA using the miRcute Plus miRNA First‐Strand cDNA Synthesis Kit (TIANGEN). RT was performed according to the manufacturers' instructions in a Mastercycler pro PCR System (Eppendorf).

Quantitative PCR was performed using the GoTaqqPCR Master Mix (Promega) and the miRcute Plus miRNA SYBR Green qPCR Detection Kit (TIANGEN) on a Roche LightCycler 480 Real Time PCR instrument (Roche), by following the manufacturers' instructions. The glyceraldehyde‐3‐phosphatedehydrogenase (*GAPDH*) gene was amplified as an internal control,^14^ and *Caenorhabditis elegans* miR‐39 (cel‐miR‐39) was amplified as an external control.^15^ The circRNAs were amplified using the following profile: 95°C pre‐degeneration for 120 seconds, 40 cycles of 95°C denature for 5 seconds, 58°C primer annealing for 30 seconds, and 72°C extension for 30 seconds. The reaction conditions of qPCR‐amplified miRNA were as follows: 95°C for 15 minutes, 5 cycles of 94°C for 20 seconds, 65°C for 30 seconds and 72°C for 34 seconds, followed by 40 cycles of 94°C for 20 seconds and 60°C for 34 seconds. All the reactions were performed in triplicates and the expression levels were calculated using the ∆Ct method.^16^ The primer sequences for the qRT‐PCR analysis have been listed in Table 1 and they were synthesized by Invitrogen.

**TABLE 1:** List of primer sequences used for the qRT‐PCR analysis

Name	Primers sequence (5′ to 3′)
Hsa_circ_0093587 F	AGAAGATGACAGGTTGCCAGT
Hsa_circ_0093587 R	TTCGAAGGTCGCCATCATTA
Hsa‐circRNA9102‐5 F	AGTGATCAGCCTTCCCACTCT
Hsa‐circRNA9102‐5 R	TTTCGAAGGTCGCCATCATT
GAPDH F	ACCCACTCCTCCACCTTTGAC
GAPDH R	TGTTGCTGTAGCCAAATTCGTT
Cel‐miR‐39‐F	TCACCGGGTGTAAATCAGCTT
Has‐miR‐150‐5p F	TCTCCCAACCCTTGTACCAGTG

### 2.6 Statistical analysis

The data have been presented in the form of mean ± standard deviation, medians (lower quartile, upper quartile) or proportional distribution according to the criterion of whether the continuous variable is normally distributed as a continuous variable or as a categorical variable. The categorical variables were analyzed with the Pearson's Chi‐square test and the continuous variables were assessed with a two‐tailed Student's *t* tests, between the two groups. Logistic regression analysis was performed to estimate whether the aberrant expression of circRNAs increased the risk of EH. Receiver operating characteristic (ROC) curves were constructed to evaluate the diagnostic value. Statistical significance was set a priori at a two‐sided *P* < .05. All data in this study were analyzed and the images were processed using the SPSS 22.0 software (SPSS Corp., Chicago, IL, USA) and the GraphPad Prism 7 (GraphPad Prism Software, La Jolla, CA, USA).

## 3 RESULTS

### 3.1 Validation of target circRNA and miRNA expression levels in blood samples by qRT‐PCR

Selected subject characteristics have been presented in the Table 2. The HC and the EH groups showed no significant differences in sex and age. However, the subjects in the control and the EH groups had significant differences in BMI (*t *= −3.723, *P* < .001) and HDL (*t* = 2.690, *P* < .008). Other than BMI, HDL, and blood pressure, there were no significant differences between the two groups in any metabolic variables. As presented in Table 2, the patients group diagnosed with EH had lower ΔCt values of hsa‐circRNA9102‐5 (11.71 ± 1.06 vs 12.13 ± 1.11, *P* = .007) as compared to the HC group. There was no statistically significant difference in the expression of hsa_circ_0093587 (11.59 ± 1.13 vs 11.77 ± 1.36, *P* = .298) between the EH and the HC groups. A higher ΔCt value indicates lower expression. This means that the expression levels of hsa‐circRNA9102‐5 were significantly higher in the EH group as compared to that in the HC group. The EH group had higher ΔCt values of hsa‐miR‐150‐5p (−0.65 ± 0.99 vs −0.93 ± 1.04, *P* = .027), indicating that the expression levels of hsa‐miR‐150‐5p were significantly lower in the EH group as compared to that in the HC group.

**TABLE 2:** Comparison of characteristics and parameters between HC and EH group

Characteristics	HC (mean ± SD)	EH (mean ± SD)	*t*/χ^2^	*P* value
Age	54.05 ± 10.56	54.36 ± 10.11	−0.210	.834
Gender (M/F)	48/48	48/48	0.000	1.000
Smoking (Y/N)	18/78	27/69	2.351	.125
Drinking (Y/N)	12/84	20/76	2.400	.121
BMI (kg/m^2^)	22.97 ± 2.70	24.49 ± 2.95	−3.723	**<.001**
SBP (mm Hg)	123.31 ± 10.80	141.74 ± 14.10	−10.167	**<.001**
DBP (mm Hg)	76.23 ± 7.73	87.54 ± 10.56	−8.467	**<.001**
HDL (mmol/L)	1.41 ± 0.29	1.31 ± 0.24	2.690	**<.008**
LDL (mmol/L)	2.86 ± 0.76	2.82 ± 0.63	0.448	.654
ALT (IU/L)	23.71 ± 14.89	25.58 ± 19.49	−0.749	.455
AST (IU/L)	22.43 ± 6.70	23.47 ± 9.36	−0.887	.376
BUN (mmol/L)	5.49 ± 1.27	5.21 ± 1.08	1.622	.106
UA (μmol/L)	360.61 ± 73.90	363.53 ± 84.40	−0.255	.799
TG (mmol/L)	1.54 ± 0.52	1.60 ± 0.74	−0.701	.484
TC (mmol/L)	5.30 ± 0.94	5.19 ± 0.80	0.855	.394
Scr (μmol/L)	75.59 ± 16.90	74.13 ± 19.01	0.566	.572
Glu (mmol/L)	5.13 ± 0.74	5.31 ± 0.87	−1.546	.124
ΔC_t_ hsa_circ_0093587	11.77 ± 1.36	11.59 ± 1.13	1.043	.298
ΔC_t_ hsa‐circRNA9102‐5	12.13 ± 1.11	11.71 ± 1.06	2.704	**.007**
ΔC_t_ hsa‐miR‐150‐5p	−0.93 ± 1.04	−0.65 ± 0.99	−2.225	**.027**

Note

Bold entries represent statistically significant values.

Abbreviations: ALT, alanine transaminsae; AST, BMI, body mass index; BUN, blood urea nitrogen; CI, confidence interval; DBP, diastolic blood pressure; EH, essential hypertension; F, female; Glu, blood glucose;HDL, high‐density lipoprotein; LDL, low‐density lipoprotein; M, male; N, no; SBP, systolic blood pressure; Scr, serum creatinine; SD, standard deviation; TC, total cholesterol; TG, triglyceride; UA, uric acid; Y, yes.

**FIGURE 1:**
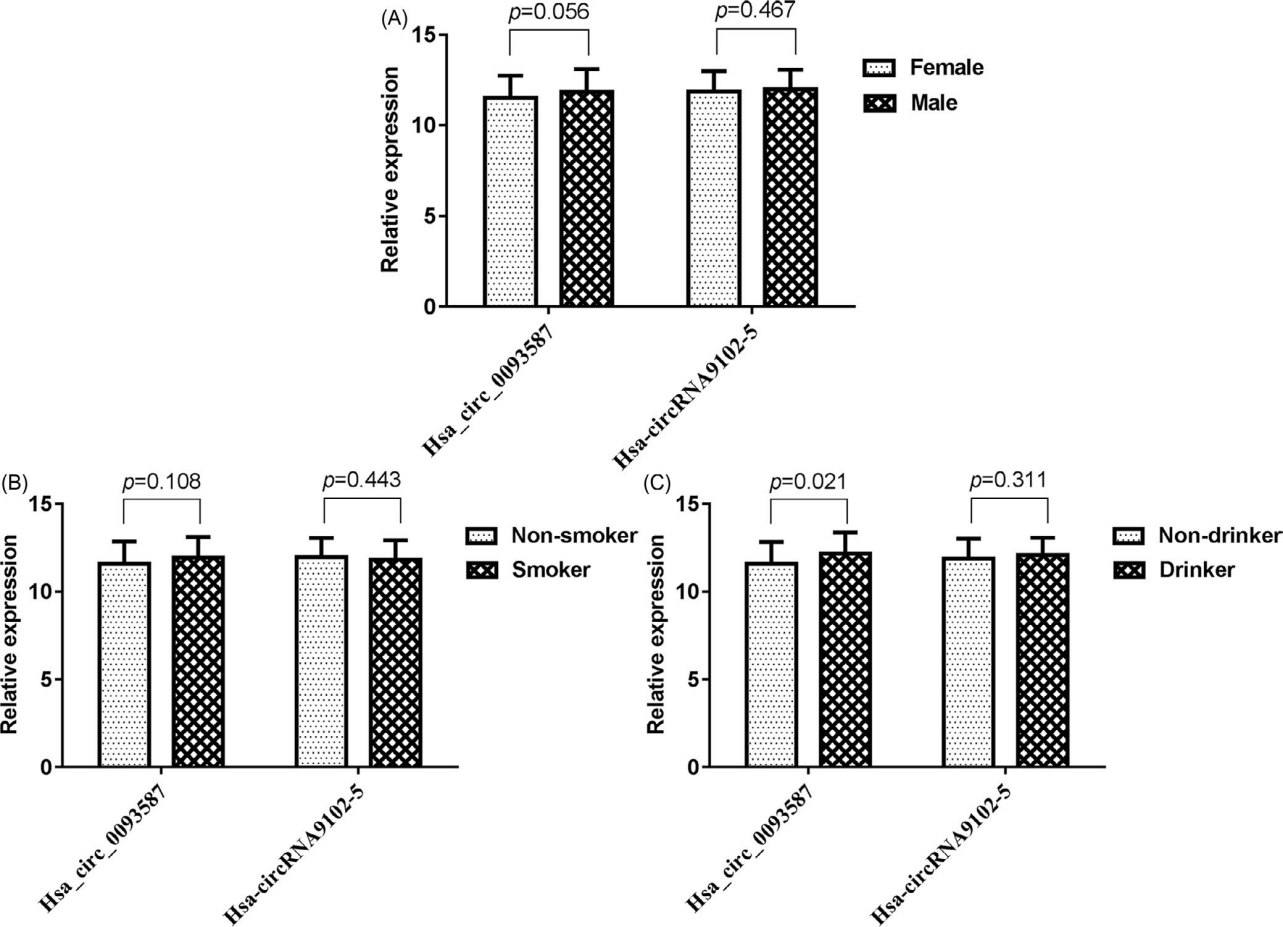
Comparison of hsa_circ_0093587 and hsa‐circRNA9102‐5 levels between (A) females and males, (B) non‐smokers and smokers, and (C) non‐drinkers and drinkers. circ, circular RNA; hsa, *Homo sapiens*

Further, we also compared the expression of circRNAs based on sex of the individual, drinking habits, and smoking habits. As presented in Figure 1, drinkers and non‐drinkers exhibited statistically significant differences in the relative expression of hsa_circ_0093587 (*P* = .021; Figure 1C), indicating that drinkers had significantly lower expression levels when compared to that in the non‐drinkers. However, both hsa_circ_0093587 and hsa‐circRNA9102‐5 were not significantly associated with sex of the individual (Figure 1A) and smoking habits (Figure 1B).

### 3.2 Factor analysis using logistic regression

After elimination of the interactions by a forward method, the results of the logistic regression analysis with *P* value adjustment showed that hsa‐circRNA9102‐5 (odds ratio = 0.555, 95% confidence interval (CI): 0.383‐0.804, *P* = .002) and hsa‐miR‐150‐5p (odds ratio = 1.523, 95% CI: 1.071‐2.164, *P* = .019) were significant predictors of EH, as shown in Table 3, which indicated that down‐regulated hsa‐miR‐150‐5p or up‐regulated hsa‐circRNA9102‐5 were important factors in the risk of EH. Furthermore, HDL (odds ratio = 0.096, 95% CI: 0.012‐0.799, adjusted *P* = .030) and BMI (odds ratio = 1.275, 95% CI: 1.109‐1.466, adjusted *P* = .001) were identified as a protective factor and a risk factor influencing EH, respectively.

**TABLE 3:** The effect of predictors for EH

Variable	OR (95% CI)	Wald	*P* value
Sex	0.570 (0.226, 1.433)	1.429	.232
Age	1.003 (0.969, 1.039	0.031	.861
BMI	1.275 (1.109, 1.466)	11.649	**.001**
Smoking	1.612 (0.559, 4.645)	0.782	.377
Drinking	2.152 (0.685, 6.763)	1.722	.189
ALT	0.984 (0.948, 1.020)	0.799	.371
AST	1.040 (0.961, 1.125)	0.966	.326
Scr	0.997 (0.973, 1.023)	0.039	.844
BUN	0.776 (0.578, 1.041)	2.859	.091
UA	0.998 (0.993, 1.003)	0.589	.443
LDL	0.952 (0.314, 2.885)	0.008	.930
TC	1.409 (0.483, 4.109)	0.395	.530
HDL	0.096 (0.012, 0.799)	4.700	**.030**
TG	0.723 (0.375, 1.395)	0.935	.334
Hsa_circ_0093587	1.033 (0.758, 1.409)	0.043	.836
Hsa‐circRNA9102‐5	0.555 (0.383, 0.804)	9.716	**.002**
Hsa‐miR‐150‐5p	1.523 (1.071, 2.164)	5.497	**.019**

Note

Statistical significant *P* values are displayed in bold. The abbreviations are the same as Table 2.

### 3.3 Functional enrichment analysis and expression level verification of target miRNAs

**FIGURE 2:**
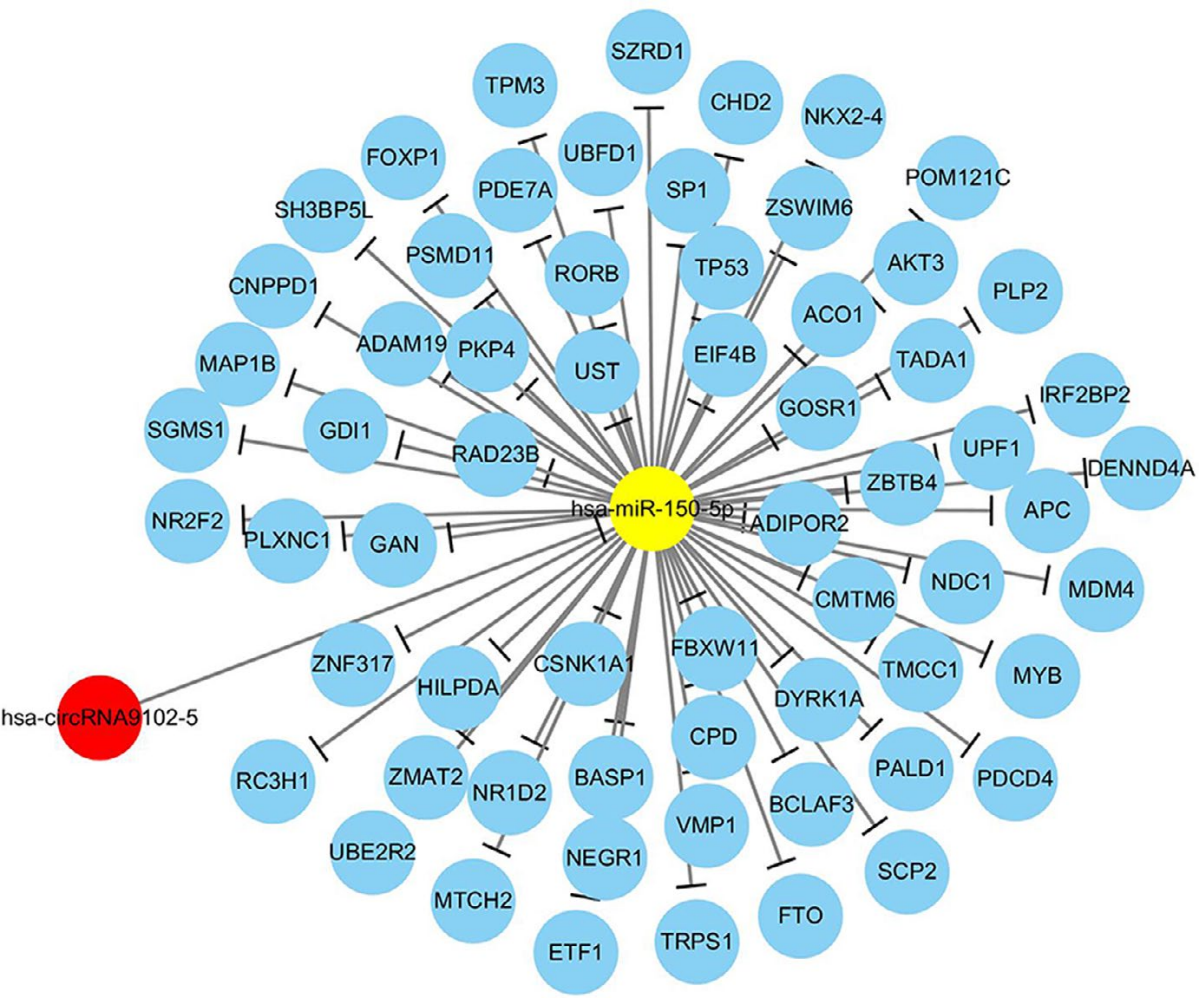
CircRNA‐miRNA‐mRNA network diagram. The red circle represents the target circRNA hsa‐circRNA9102‐5, the blue circle represents the miRNA, and the yellow circle represents the target miRNA hsa‐miR‐150‐5p. The lines represent the connection and binding of each other

**FIGURE 3:**
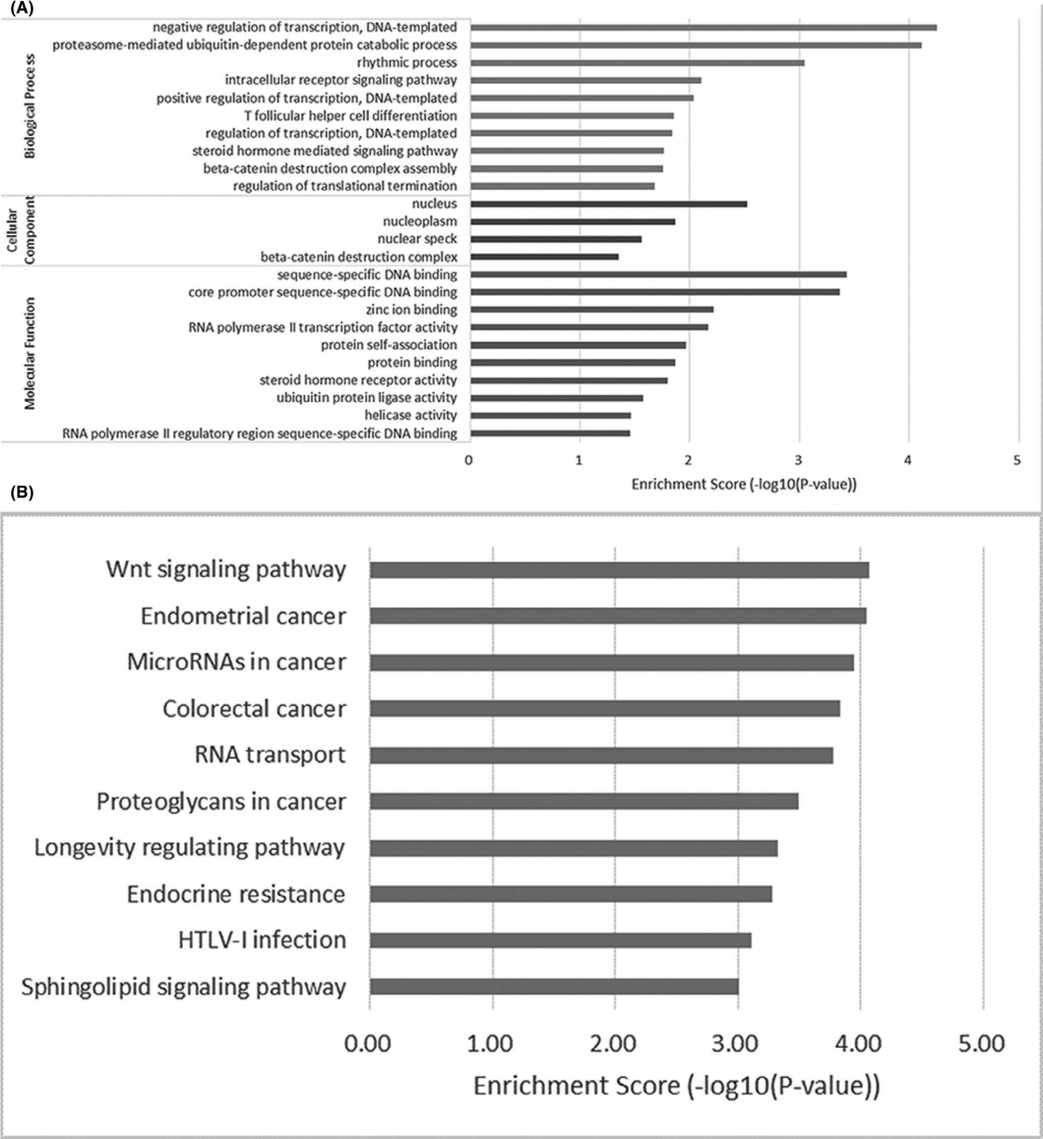
A presents GO analysis of hsa‐miR‐150‐5p. B presents KEGG analysis of hsa‐miR‐150‐5p

Considering the intersection of the predicted results of three bioinformatics databases, we found that hsa‐miR‐150‐5p had 63 mRNAs. As shown in Figure 2, the network diagram of circRNA‐miRNA‐mRNA was illustrated. According to the GO analysis, the steroid hormone‐mediated signaling pathway (*P* = .017) was enriched in the biological process of hsa‐miR‐150‐5p as shown in Figure 3A. Based on the KEGG analysis, the Wnt signaling pathway was the most enriched pathway (*P* = 8.42E−05) in the KEGG analysis of hsa‐miR‐150‐5p, followed by several cancer pathways in endometrial cancer (*P* = 8.90E−05) and colorectal cancer (*P* = 1.47E−04) as presented in Figure 3B.

### 3.4 Diagnostic accuracy of circRNAs and miRNAs as biomarkers

**FIGURE 4:**
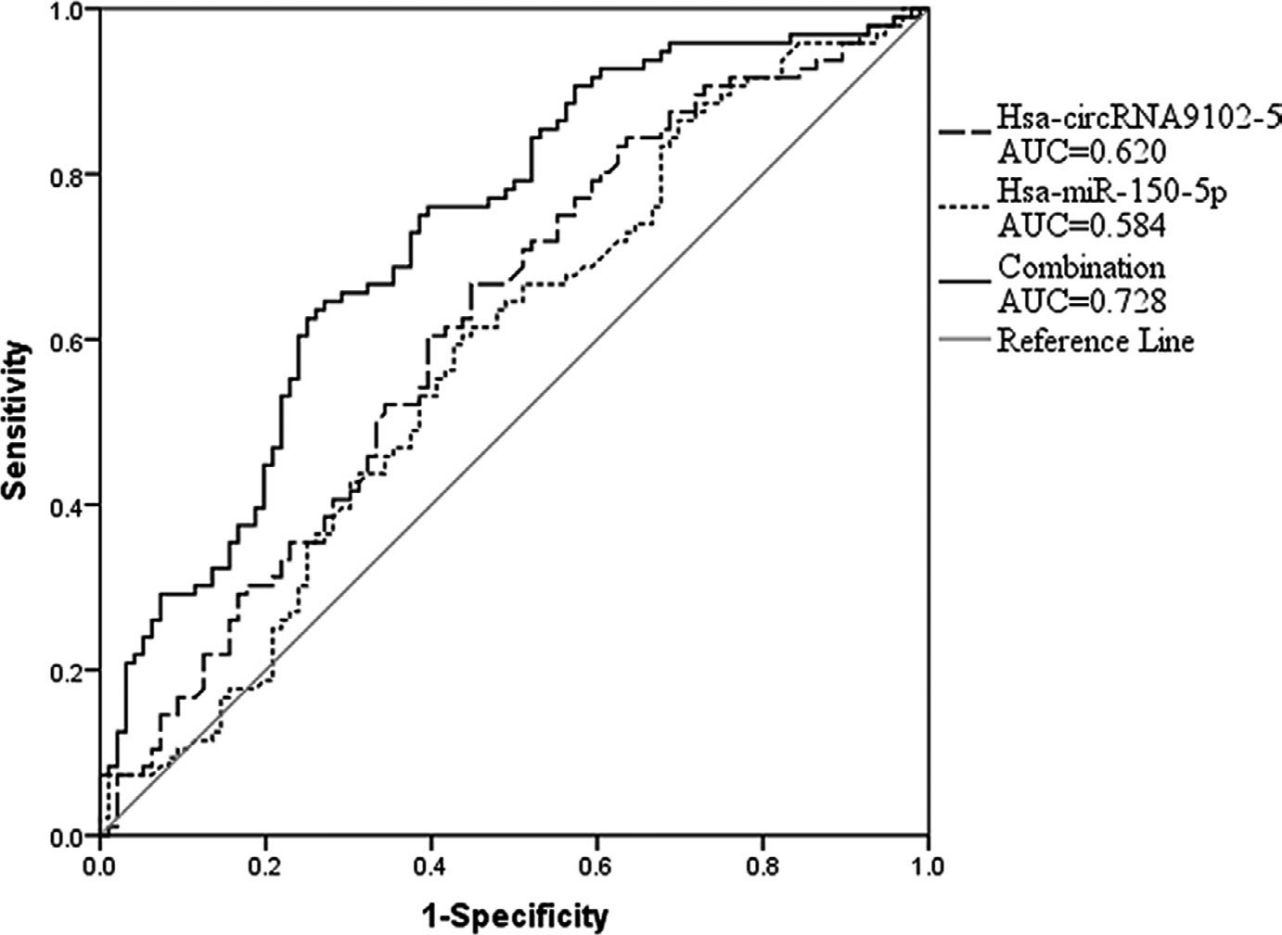
The ROC curve analysis for hsa‐circRNA9102‐5, hsa‐miR‐150‐5p and combination

To further evaluate the accuracy of these circRNAs and miRNAs as candidate biomarkers in the diagnosis of EH, the ROC curves were generated (Figure 4). The area under the curve (AUC) for hsa‐circRNA9102‐5 was 0.620 (95% CI = 0.541‐0.699, *P* = .004). The sensitivity and specificity were 0.667 and 0.552, respectively. The AUC for hsa‐miR‐150‐5p was 0.584 (95% CI: 0.503‐0.665, *P* = .045), and the sensitivity and specificity were 0.594 and 0.573, respectively. To improve the diagnostic value of the biomarkers, we combined hsa‐miR‐150‐5p with hsa‐circRNA9102‐5, associated them with the environmental risk factors of EH (BMI, smoking and drinking), and then tested the combined indicator as a new biomarker using ROC analysis. The result showed that the AUC reached 0.728 (0.657‐0.799, *P* < .001), and the sensitivity and specificity were 0.646 and 0.739, respectively. The above results implied that compared to a single circRNA type, the combined indices of multiple factors may have a higher diagnostic value.

## 4 DISCUSSION

Peripheral blood circRNAs have been reported to be differentially expressed in many diseases and many studies have confirmed that it can be used as a biomarker for disease diagnosis.^17^, ^18^ CircRNAs serve as miRNA sponges by binding to a variety of miRNA recognition elements (MREs), which can promote the expression of downstream target genes by competitively binding miRNAs, and play an important role in miRNA‐mediated post‐transcriptional gene regulation.^19^ However, only a few studies have reported the circRNA‐related roles in EH. This study attempted to explore the role of circRNAs in the pathogenesis of EH and their potential as a biomarker.

Based on a bioinformatics predictive analysis, we found that hsa‐circRNA9102‐5 had several binding sites of hsa‐miR‐150‐5p. MiR‐150, and at low circulating levels, were associated with many CVD and vascular events, eg, miR‐150 has been associated with cardiovascular protective effects.^20^ Indeed, Karolina et al^21^ found that miR‐150 presented low expression levels in the blood of the patients with hypertension as compared to that in the healthy controls. MiR‐150 can promote angiogenesis and endothelial cell migration in vitro and in vivo by acting on the vascular endothelial cells.^22^ Researchers have also suggested that miR‐150 may promote the proliferation and the migration of endothelial cells and may also maintain the function of endothelial cells by inhibiting the expression of PTX3.^23^ It has been hypothesized that endothelial dysfunction and decreased angiogenesis, caused by a low expression level of miR‐150, may be closely related to the occurrence of hypertension. Further in this study, results showed that the steroid hormone‐mediated signaling pathway was enriched in the GO enrichment analysis. Aldosterone, an important steroid hormone, has been reported to be directly involved in the regulation of blood pressure by regulating water content, salt metabolism, and blood volume.^24^ For the above‐mentioned reasons, the circulating vascular‐related hsa‐miR‐150‐5p was considered as the target miRNA for hsa‐circRNA9102‐5, in the study.

Through the KEGG signaling pathway analysis, the Wnt signaling pathway was found to be most significantly enriched in the pathway analysis for hsa‐miR‐150‐5p. Recent studies have found that the Wnt signaling pathway affects the peripheral regulation and the central regulation of blood pressure, and the VSMC remodeling caused by Wnt signal disturbance may be the basis for the increase of blood pressure.^25^ Our results showed that the expression level of hsa‐miR‐150‐5p in the peripheral blood was lower in the EH group compared to that in the HC group, which was contrary to the trend of hsa‐circRNA9102‐5 up‐regulation. Combined with the above‐mentioned analysis, their function as protective factors in regulating human blood pressure has also been demonstrated. Thus, it is reasonable to hypothesize that hsa‐circRNA9102‐5 may participate in the regulation of EH by inhibiting the function of hsa‐miR‐150‐5p.

We also estimated the diagnostic value of selected circRNAs and miRNAs in EH by establishing the ROC curve for differentiating EH patients from the HC. Among these RNAs, hsa‐circRNA9102‐5 had the largest AUC of 0.620, and its sensitivity and specificity were 0.667 and 0.552, respectively. Compared to an independent predictor, the AUC of a new biomarker, consisting of combined multiple factors, increased to 0.728, which indicated an improvement in the diagnostic value. It is not difficult to infer that in this study, circRNAs had a certain diagnostic value for EH, but it was not sufficient to be applied in clinical diagnosis. We suggest that circRNA differential expression, as per this study, may not be sufficient to replace the current method for blood pressure measurement and may not be as convenient as the latter, but it may be used for the distinction and identification of special types of hypertension, which will require further research and confirmation.

Our present study is the first to demonstrate that hsa‐circRNA9102‐5 expression is up‐regulated in EH. We also found statistically significant differences in BMI and HDL between the EH and the HC groups, and the subsequent regression analysis indicated that BMI and HDL were risk factor and protective factor of EH, respectively, consistent with our general cognition. However, our present study had some limitations. It was a single‐center study, and all the subjects were geographically concentrated in one city. Clarification will be needed whether selected RNAs in other populations show the same differential expression as reported in this study. Therefore, the conclusions of this study will require further verification in a multi‐center cohort with a larger sample size. Additionally, though bioinformatics techniques were used to predict the binding sites between circRNAs and target miRNAs, hsa‐circrna9102‐5 as a sponge of hsa‐mir‐150‐5p needs further confirmation. Our group will further complete the experiment such as RNA immunoprecipitation and double luciferase reporter gene experiment in our future studies.

In conclusion, the present study was the first to demonstrate that the expression levels of hsa‐circRNA9102‐5 was up‐regulated in EH. Additionally, it may be hypothesized that hsa‐circRNA9102‐5 may act as a specific sponge for hsa‐miR‐150‐5p to participate in the regulation of EH, as determined by qRT‐PCR validation and bioinformatics analysis. In this study, hsa‐circRNA9102‐5 had a certain diagnostic value for EH as an independent biomarker, and the diagnostic value was increased after introducing multiple other factors.
